# Oh, the places you will grow: Intraspecific latitudinal clines in butterfly size suggest a phylogenetic signal

**DOI:** 10.1002/ece3.8913

**Published:** 2022-05-19

**Authors:** Andrew C. Merwin, Justin Hilliard, Ashley Larsen, Andrew G. Lasken, Icesstrená Johnson

**Affiliations:** ^1^ 1651 Department of Biology and Geology Baldwin Wallace University Berea Ohio USA; ^2^ Citizens for Los Angeles Wildlife Los Angeles California USA

**Keywords:** Bergmann's Rule, latitudinal clines, Lycaenidae, Riodinidae, host plant nitrogen

## Abstract

Within an animal species, the body sizes of individuals at higher latitudes are often different from individuals at lower latitudes. For homeothermic species that maintain a relatively constant body temperature, such as mammals and birds, individuals at higher latitudes tend to be larger. For ectothermic species, such as insects, that do not retain their own body heat and which often do not maintain a relatively constant body temperature, patterns of body size with latitude are highly variable. This has led some authors to contend that patterns in even closely related species cannot be expected to be similar. Indeed, to our knowledge, no studies of invertebrates have found that more closely related species have more similar relationships between body size and latitude. Further, no studies have investigated the potential influence of diet quality on interspecific differences in these clines. We measured wing lengths of specimens (N = 1753) in eight lycaenid butterfly species and one species of the sister family, Riodinidae to determine if more closely related species have similar latitudinal trends. We also estimated the mean nitrogen content of caterpillars’ hosts to investigate whether this often‐limiting nutrient influences the strength and direction of latitudinal clines in body size. We found that four species are significantly smaller at higher latitudes, an additional species is marginally smaller at higher latitudes (*p* < .06), and four species had no significant relationship with latitude. We also found a strong phylogenetic signal for latitudinal clines in body size among our species, which indicates that some closely related species may have similar clines. However, the strength and direction of these clines did not depend on the estimated nitrogen content of caterpillars’ hosts. Our results indicate that mean nitrogen content of hosts may not be an important driver in latitudinal clines but that phylogenetic relationships among species should be accounted for when exploring other potential drivers of body‐size clines in invertebrate species.

## Graphical abstract

Latitudinal gradients in invertebrate body size vary widely. Yet, in our study we found that more closely related butterfly species have more similar latitudinal gradients. This research suggests a species‐level inheritance for sensitivity to factors associated with latitude and underscores the necessity of accounting for relatedness among species when attempting to use comparative approaches to investigate mechanisms underlying latitudinal trends in invertebrate body‐size.

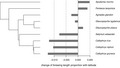

## 1 INTRODUCTION

Within an animal species, the body sizes of individuals at higher latitudes are often different from individuals at lower latitudes. For example, homeothermic animals, like mammals and birds, tend to be larger at higher latitudes than they are at lower latitudes (Ashton, 2002; Ashton et al., 2000). Bergmann originally proposed that larger body sizes at higher latitudes are adaptive and facilitate the retention of body heat in colder climates through a reduction in surface area‐to‐volume ratio (1847, as referenced in Salewski & Watt, 2017). Although the mechanisms responsible for the observed trend of increasing body size with latitude are still debated (e.g., Angilletta et al., 2004; Ashton et al., 2000), positive relationships between body size and latitude—so‐called Bergmann clines—are, nevertheless, frequently observed for homeothermic species. Among ectothermic species*—*those that do not retain their own metabolic heat—relationships between body size and latitude are far more variable (Adams & Church, 2008; Ashton & Feldman, 2003; Blanckenhorn & Demont, 2004; Horne et al., 2015; Shelomi, 2012). For instance, insects—which comprise the largest class of ectothermic species—appear to have nearly an equal likelihood of exhibiting Bergmann clines, converse‐Bergmann clines (negative relationships between body size and latitude), or no discernible relationship between body size and latitude (Shelomi, 2012).

Many nonmutually exclusive explanations have been proposed to account for relationships between body size and latitude in insects—most of which depend on plastic developmental responses to abiotic conditions. For instance, body size in aquatic insects seems to generally increase with latitude, perhaps because dissolved oxygen concentration—a limiting factor to development in aquatic environments—is greater in cold water (Horne et al., 2015). By contrast, terrestrial insects—especially those with one generation a year—tend to have negative relationships between body size and latitude (Horne et al., 2015). This observation is consistent with expectations for a tradeoff between body size and season length, whereby larger body sizes are achieved in terrestrial insects at lower latitudes where the growing seasons are longer. Other explanations for variation in latitudinal patterns in insect body size are less congruous. For instance, Tseng and Soleimani Pari (2019) provide empirical evidence that beetles with greater mean body sizes may be under more selection pressure to retain larger body sizes for thermoregulation and, therefore, may be less likely to exhibit converse‐Bergmann clines. By contrast, Blanckenhorn and Demont (2004) review latitudinal clines across Arthropoda and suggest that at large taxonomic scales, larger‐bodied arthropods may be more likely to exhibit converse‐Bergmann clines (Blanckenhorn & Demont, 2004).

These general and sometimes contrasting explanations highlight the many and varied mechanisms underlying changes of body‐size with latitude and suggest that much variation remains unexplained among insects. For example, two important and underexplored drivers of latitudinal trends in body size are evolutionary relatedness and diet. In fact, to our knowledge only one study (Miller & Sheehan, 2021) has explicitly investigated whether closely related species (congeners) have similar latitudinal clines in body size and no studies have attempted to relate the strength of latitudinal clines among species to their mean host quality (host plants or prey species).

Some studies have found such idiosyncratic patterns of insect body size with latitude that their authors contend that clines in one species cannot be expected to reflect those of even confamilial or congeneric species (Shelomi, 2012; Shelomi & Zeuss, 2017). Supporting this assertion, Shelomi and Zeuss (2017) found near‐Bergmann clines and converse‐Bergmann clines among confamilial species of stick insects in Europe. Further, Sota et al. (2000) found significant Bergmann and converse‐Bergmann clines within the same genus of ground beetles. Yet, to our knowledge, Miller and Sheehan (2021) is the only study to explicitly test for a phylogenetic signal—that is, whether more closely related species have more highly correlated latitudinal clines in body size. When coding latitudinal clines in body size as discretely positive or negative, they found that paper wasps (*Polistes* spp.) of North America exhibited no phylogenetic signal; several species exhibited Bergmann clines and many exhibited converse‐Bergmann clines with multiple origins indicated for both positive and negative clines. Whether or not other insect taxa exhibit phylogenetic patterns in the strength and direction of their relationships between body size and latitude, however, remains unknown. Uncovering such phylogenetic signals in intraspecific latitudinal clines would indicate a heritable, species‐level response to latitude (De Queiroz & Ashton, 2004) and could carry implications for the generation of more mechanistic hypotheses to explain variability in body‐size clines with latitude in insects.

Nutritional differences among insects’ host species—particularly in nitrogen content—could also contribute to observed interspecific differences in the slope of latitudinal clines. For instance, many insect herbivores are nitrogen limited (Lavoie & Oberhauser, 2004; Mattson, 1980), and evidence from intraspecific studies indicates that insects feeding on hosts that are low in nitrogen typically develop more slowly and achieve smaller adult body sizes (Teder et al., 2014). For this reason, closely related insects feeding on hosts that are generally lower in nitrogen content may spend more time feeding and developing and may, therefore, be more sensitive to reduced season lengths in higher latitudes. If this were the case, one might hypothesize that insects specialized to feed on lower‐nitrogen host plants may have stronger declines in body size with increasing latitude. Although mean host nitrogen content could potentially influence the strength of intraspecific latitudinal clines in body size, to our knowledge, this has not yet been investigated.

Here, we report on a study investigating the body size‐latitude relationships among a small sample of North American butterflies: eight species of Lycaenidae (Lepidoptera, Arthropoda) and one species in the sister family Riodinidae. We used extensive digitized museum collections to ask three questions:

1. What geographic and bioclimatic variables (mean annual precipitation and temperature, elevation, longitude, and latitude) help to explain variation in body size?
2. Do latitudinal clines depend on phylogenetic relatedness? And
3. Accounting for evolutionary relationships among species, do species’ linear slopes of adult body size with latitude depend on mean nitrogen content of caterpillar species’ diet?

## 2 MATERIALS AND METHODS

### 2.1 Species and specimens

We chose to study eight species in the family Lycaenidae from four subfamilies and one species from the sister family Riodinidae (Table 1). These species were selected for four reasons. First, we were interested in studying species that are nonmigratory and have a relatively small lifetime range so that available geographic and bioclimatic data could be more precisely associated with available specimens’ location of collection. Second, we selected species that had a relatively broad latitudinal range (>10°), so we could examine latitudinal clines. Third, we chose species that exhibit wide variation in host nitrogen content, including one species, *Feniseca tarquinius*, which is carnivorous as a caterpillar. Finally, we selected species for which more than 100 specimens were measurable through digital images on the Global Biodiversity Information Facility (GBIF.org, 2020). Our nine chosen species capture a small proportion of the approximately 139 species of lycaenids and 20 species riodinids in North America. Thus, some caution should be applied when interpreting our results across these families.

**TABLE 1:** Butterfly species used in our study, including the taxonomic affiliations of each species, number of generations in our study, the sample size, and the common host families of each butterfly species

Species	Family	Tribe	Gen.	Sample
*Apodemia mormo*	Riodinidae	Emesiini	1^a^	211
*Feniseca tarquinius*	Lycaenidae	Spalgini	2–3	104
*Agriades glandon*	Lycaenidae	Polyommatini	1	122
*Glaucopsyche lygadmus*	Lycaenidae	Polyommatini	1	645
*Glaucopsyche piasus*	Lycaenidae	Polyommatini	1	104
*Satyrium edwardsii*	Lycaenidae	Eumaeini	1	135
*Callophrys irus*	Lycaenidae	Eumaeini	1	173
*Callophrys niphon*	Lycaenidae	Eumaeini	1	142
*Callophrys gryneus*	Lycaenidae	Eumaeini	2	116

^a^ *Apodemia mormo* has one brood throughout most of its range, although some rare biotypes in the south may have multiple broods (Pratt & Ballmer, 1991).

### 2.2 Data collection from digitized specimens

**FIGURE 1:**
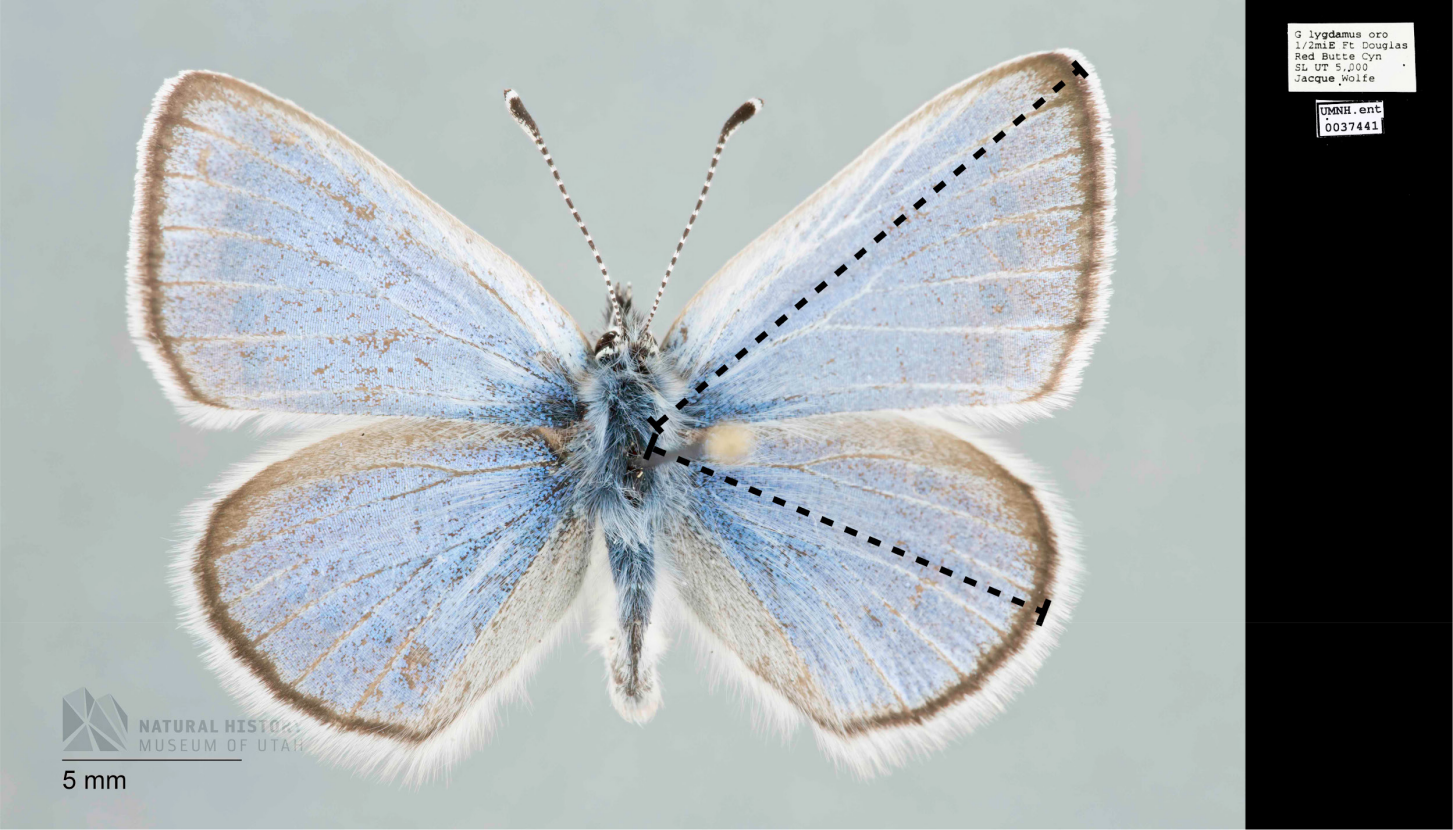
Example butterfly wing measurements on *Glaucopsyche lygdamus*. Dashed lines show the wing length measurements for forewings (top) and hindwings (bottom). (Photo UMNH ENT 0037441, Courtesy of Natural History Museum of Utah UMNH)

Wing length (used as a proxy for body size; García‐Barros, 2015; Nylin & Sviird, 1991) and location of collection were obtained from digitized specimens accessed through the GBIF portal. We measured undamaged wings using PixelZoomer (Matthias Schuetz, Darmstadt, Germany) for all images of specimens with at least one undamaged wing in the same plane as a scale bar. Length was measured from the point of wing attachment to the margin of the wing along the longest chord (Figure 1). When both of a pair of wings met our criteria, their lengths were averaged. The majority of specimens had latitude and longitude data on the specimen label. For 245 specimens that lacked latitudinal and longitudinal data, we used the centroid of the smallest geographic municipality listed, provided that it was smaller than the state or province level. We looked for potentially misleading specimen locations by (1) verifying that the coordinates corresponded correctly to their listed state or province and by (2) visually comparing our specimens’ locations to the corresponding species’ ranges provided in Scott (1992). We thereby removed two *C*. *niphon* specimens from our dataset that were listed in California, USA—far outside their range. As a result of the uneven distribution of available specimens across their range, the northern‐most *C*. *niphon* specimen is far apart from other available specimens. This particular specimen, if removed, does not change the significance of our analyses.

**FIGURE 2:**
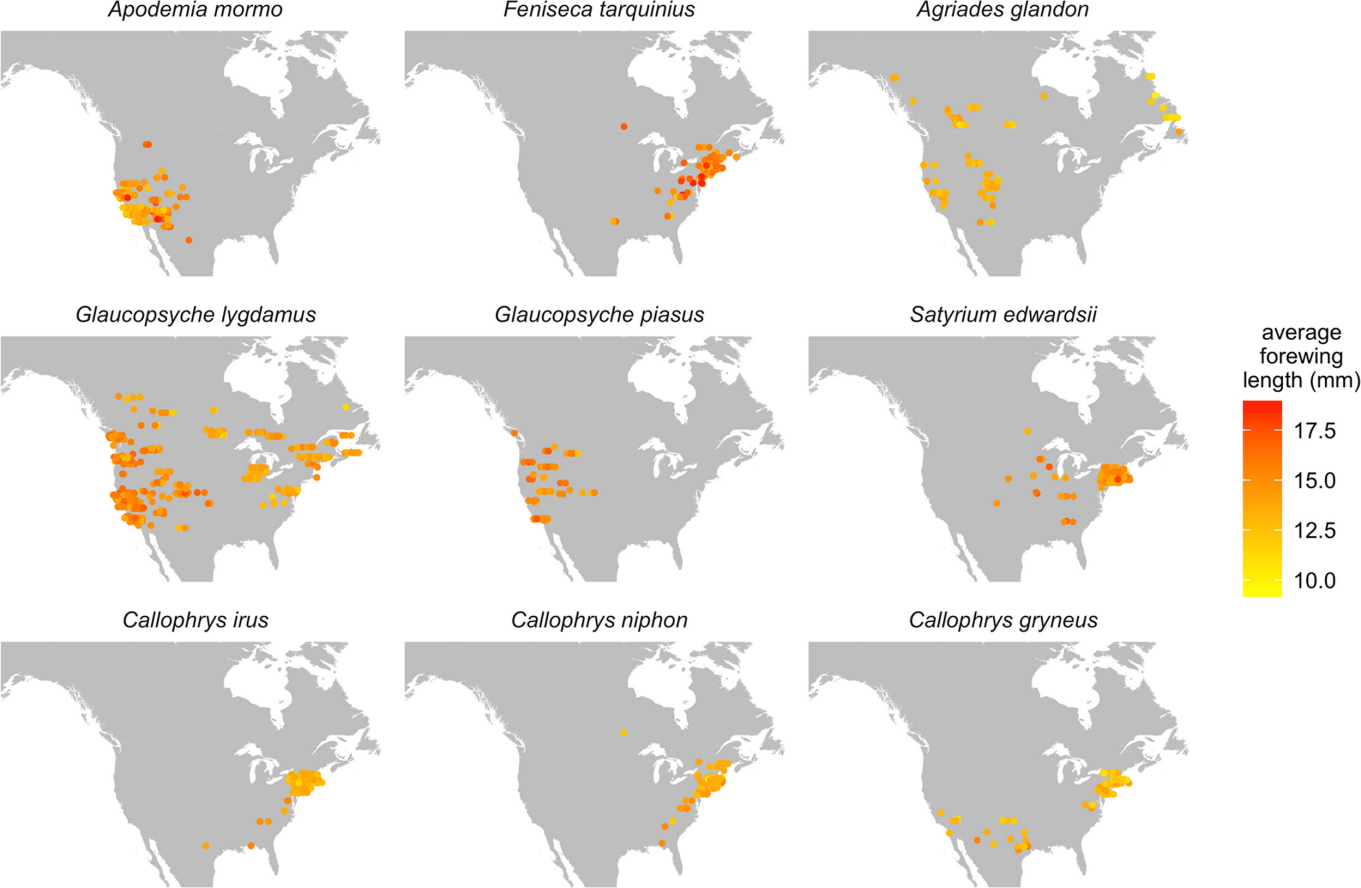
Specimen locations for the nine species of butterflies used in this study. Points are slightly jittered along the horizontal axis to reduce overlap

In total, our analyses included 1753 measured individuals from across North America and Canada (Figure 2). Early analyses of hindwings revealed qualitatively similar patterns to analyses with forewings, including a significant phylogenetic signal and, for simplicity, are not discussed. In addition, we were unable to test for the presence of differences between sexes due to the lack of overt sexual dimorphism among most species and because sex was generally not indicated on label information.

### 2.3 Geographic and bioclimatic and host nitrogen data

Elevation, mean annual precipitation, and mean annual temperature for the latitude and longitude of each specimen were collected to test for the relative importance of these geographic and bioclimatic variables in determining body size. Elevation data were retrieved from the Shuttle Radar Topography Mission at a resolution of 90m x 90m through the Global Biodiversity Information Facility (package ‘rgbif’; Chamberlain & Boettiger, 2017). Mean annual precipitation (mm) and temperature (°C) from 1970 to 2000 were retrieved through the World Clim 2 database (Fick & Hijmans, 2017) at a resolution of 2.5 arc minutes. (~4.6 km).

In addition to determining the relationship between latitude and body size, we were interested in examining a possible relationship between mean nitrogen content of species’ larval hosts and their latitudinal clines in body size. To investigate this possible relationship, we used Scott (1992) to determine the common host plants of each species. We then used data available through the TRY Plant Trait Database (Kattge et al., 2020) to identify the estimated nitrogen content of each plant. In some instances, where nitrogen values for host plant species were unavailable, we substituted the host plant species with a related species of the same genus. When multiple nitrogen values were available from multiple specimens of the same host plant species, we used the mean of those values. For species that fed on multiple host plants, we used the mean of mean nitrogen contents of all available species. One of our butterfly species, *Feniseca tarquinius*, is carnivorous in its larval stage, and we used the nitrogen content of insects of the same family as its prey (Aphididae) from Fagan et al. (2002).

### 2.4 Phylogenetic analysis and chronogram construction

COI sequences were retrieved from GenBank for all species except *Satyrium edwardsii*, for which we substituted a congener, *Satyrium w‐album*. An alignment was made using the MUSCLE algorithm (Edgar, 2004) as implemented in MEGA X (Kumar et al., 2018) using default settings. Jalview v2.11.0 (Waterhouse et al., 2009) was used to extract a consensus sequence for *A*. *mormo*.

Maximum Likelihood (ML) phylogenies were reconstructed for tribes Eumaeini and Polyommatini separately, using the *A*. *mormo* consensus as the outgroup for both. IQ‐TREE v2.0 was used for tree reconstruction with automatic model selection via ModelFinder (Kalyaanamoorthy et al., 2017) and branch support was determined using nonparametric bootstrapping (Felsenstein, 1985). The resulting ML tree was rescaled using the root date of 89.41mya, the last common ancestor (LCA) for Riodinidae (*A*. *mormo*) and Lycaenidae (Eumaeini and Polyommatini) as determined by the chronogram in Espeland et al. (2018).

A final tree was manually constructed in R using the “phytools” package (Revell, 2012), grafting the reconstructed Eumaeini and Polyommatini trees to a tree with *A*. *mormo* and *F*. *tarquinius*. The time calibrations for the LCA of *F*. *tarquinius* and the Eumaeini/Polyommatini clade and the LCA for Eumaeini and Polyommatini were determined using the chronogram in Espeland et al. (2018). The R Script and data used (alignments and phylogenetic trees) are available in the Online Supplement.

### 2.5 Statistical analyses

Unless stated otherwise, all analyses were conducted in R v. 4.1.2 (R Core Team, 2020) in the RStudio environment (RStudio Team, 2020).

#### 2.5.1 Geographic and bioclimatic variable importance

The geographic and bioclimatic variables we used are inherently multicollinear. For this reason, we assessed variable importance using conditional random forests (Breiman, 2001; Strobl et al., 2009), which, unlike more traditional parametric statistics, are particularly insensitive to covariation among variables. Models were fit for each species in R with the “party” package (Hothorn et al., 2006; Strobl et al., 2007, 2008). For hyperparameter tuning, *mtry* was set at 3 and the tree number tuned until variable importance stabilized at random seeds of 37 and 72 (15,000 trees for *S*. *edwardsii*; 7000 trees for *G*. *lygdamus*, *C*. *niphon*, and *A*. *glandon*; 5000 trees for *C*. *gryneus*; and 3000 trees for all other species; Strobl et al., 2008). The results were visualized with a bubble plot. Data and an example R script are available in Merwin et al. (2022).

Accumulated Local Effects (ALE) plots were used to visualize the relationships between every species’ forewing length and the covariates individually. Briefly, quantiles are used to divide the covariate into intervals with equal sample numbers. The effect of each interval is calculated and compared against the overall average prediction, resulting in the ALE value for that interval. Every point represents the center of an interval and its associated ALE value. See Molnar (2019) for further reading. ALE plots are in the Supporting information.

#### 2.5.2 Estimates of linear latitudinal gradients of body size

To estimate the linear change in body size with latitude, we built generalized additive mixed‐effects models (GAMMs) for each species in R with the “mgcv” package (Wood et al., 2016). These models included a smooth term for longitude to account for changes in body size that could occur due to east–west patterns. Mean annual precipitation and temperature were not included in the estimates of latitudinal gradients due to their strong multicollinearity with latitude. Further, elevation was excluded because it was not significant in any of the species models. To make conservative estimates of significance and to account for correlations in body size among individuals collected at the same location, we also included the site of collection as a random effect. The models for each species took the following formula:

$$\begin{array}{c} \text{for ewing size}\;∼\;\text{latitude}\;+\;s\left(\text{longitude}\right)\;+\;\left(1|\text{collection coordinates}\right) \end{array}$$

Where *s(longitude)* indicates a smooth term for longitude and (*1|*collection coordinates) indicates the random intercept of collection site.

Estimated slopes of the body size–latitude relationship for each species were then divided by the mean body size of the species to provide an estimate of the proportion of change with degree latitude. We did this to follow established precedent (e.g., Tseng & Soleimani Pari, 2019), however, using change in absolute wing size (mm) with latitude yielded similar and significant results (results not shown).

#### 2.5.3 Phylogenetic signal of linear latitudinal clines of body size

Using the estimated slope of the proportion of wing length change with latitude (from the GAMMs) along with our phylogenetic tree, we tested if more closely related species share similar latitudinal clines of body size. With the “phylosignal” package (Keck et al., 2016), we tested for phylogenetic autocorrelation in slopes of relative wing length with latitude among species using five different metrics: Moran's I (Moran, 1950), Abouheif's *C*_mean_ (Abouheif, 1999) Blomberg's K and K* (Blomberg et al., 2003) and Pagel's λ (Pagel, 1999).

To thoroughly investigate the potential for a “false positive,” we also performed a simulation in which we constructed a large, random phylogenetic tree with 159 tips (reflecting the estimated total number of Riodinidae and Lycaenidae species in North America), randomly assigned trait values ranging between −0.17 and 0.7 to each tip (reflecting the range of values observed for latitudinal clines in our study), and tested for a phylogenetic signal. We repeated this test 10,000 times. Of these tests, 459 had significant phylogenetic signals (Bloomberg's K *p < .05), indicating the probability for “false positives” (i.e., type 1 errors) is .0459 and approximately .05—the conventionally accepted cutoff. Thus, we feel confident in accepting a significant phylogenetic signal in our study.

#### 2.5.4 Influence of mean host nitrogen content on linear latitudinal clines of body size

To test for an influence of mean host nitrogen content on the sensitivity of butterfly species to changes in body size with latitude, while also accounting for relatedness among species, we used a phylogenetic generalized least squares regression (function *pgls*, package “caper,” Orme et al., 2018) with the slope of each species’ latitudinal cline as the response variable and their average host nitrogen content as the dependent variable. This model is comparable to a linear regression with nine points, one for each species, where each point describes the combination of the slope of the latitudinal gradient and mean nitrogen content across all host plants for that species. However, unlike a traditional linear regression, the phylogenetic generalized least squares regression accounts for the nonindependence among species due to their shared evolutionary history. Because we were primarily interested in the effects of host plant nitrogen on latitudinal gradients and due to our relatively small sample size (nine species), we did not include other butterfly traits (e.g., voltinism) or bioclimatic variables in this model.

## 3 RESULTS

### 3.1 Geographic and bioclimatic variable importance

**FIGURE 3:**
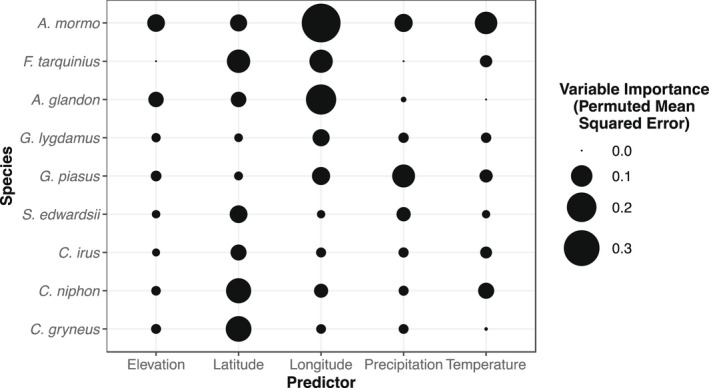
Conditional random forest importance plots with species models organized (rows) and explanatory variables (columns). Variable importance was measured as permutation importance of each factor, or the average effect on mean squared error when the factor is removed. Importance is scaled by size with higher importance equaling higher error when that factor is removed from the model. Any instance with a negative value, indicating that the randomly permuted data was better, was forced to zero for plotting purposes. The raw numbers can be found in the Online Supplement

For five of our nine species, latitude was the most important factor explaining differences in forewing length (Figure 3), precipitation was most important for *G*. *piasus*, and longitude was the most important factor for the remaining three species. Mean annual precipitation, mean annual temperature, and elevation were generally less important for most species and had largely idiosyncratic relationships with forewing length (Figures S3–S5), although mean annual temperature was of considerable importance for explaining body sizes of *Apodemia mormo* (importance = ~0.11). Of particular note, there is a shift from latitude having highest importance in Eumaeini to longitude having highest, or near highest, importance in all other taxa.

### 3.2 Linear estimates of latitudinal clines

**FIGURE 4:**
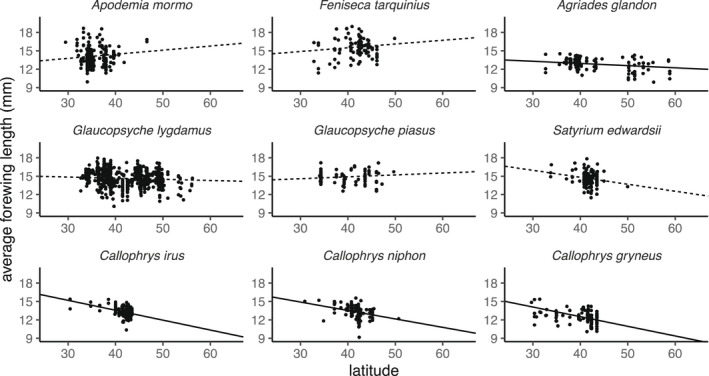
Scatterplots of average forewing length (mm) with latitude for each butterfly species. Lines indicate the estimated mean relationship between latitude and forewing size. Solid lines indicate significant relationships (*p* < .05), while dashed lines are marginally significant (*p* < .06; *S*. *edwardsii*) or insignificant

Latitudinal clines in body size ranged from slightly—though insignificantly—positive in *Apodemia mormo* and *Feniseca tarquinius* to strongly and significantly negative for all Eumaeini (Table 2), with the exception of *Satrium edwardsii*, which was only marginally significant (*p* = .0525). The negative relationships for Eumaeini were corroborated by the random forest ALE plots (A1). Species in Polyommatini exhibited much weaker body size‐latitude relationships, though *Agriades glandon* exhibited a weak, yet significant, negative relationship (Figure 4).

**TABLE 2:** Model results for GAMMs analyzing the influence latitude on forewing size for each butterfly species

Species	Change in mm with latitude	SE	*df*	*t*‐value	*p*‐value
*Apodemia mormo*	0.067	0.057	56	1.171	.246
*Feniseca tarquinius*	0.061	0.070	55	0.877	.384
*Agriades glandon*	−0.034	0.014	52	−2.455	.019*
*Glaucopsyche lygdamus*	−0.019	0.012	169	−1.515	.132
*Glaucopsyche piasus*	0.031	0.028	21	10.101	.283
*Satyrium edwardsii*	−0.114	0.057	51	−1.985	.053**
*Callophrys irus*	−0.161	0.069	36	−2.329	.026*
*Callophrys niphon*	−0.138	0.044	47	−3.148	.003*
*Callophrys gryneus*	−0.158	0.060	39	−2.623	.012*

Note

Models include a smooth term for longitude and a random effect of collection site. *p < .05; **p < .10.

### 3.3 Phylogenetic signal

**FIGURE 5:**
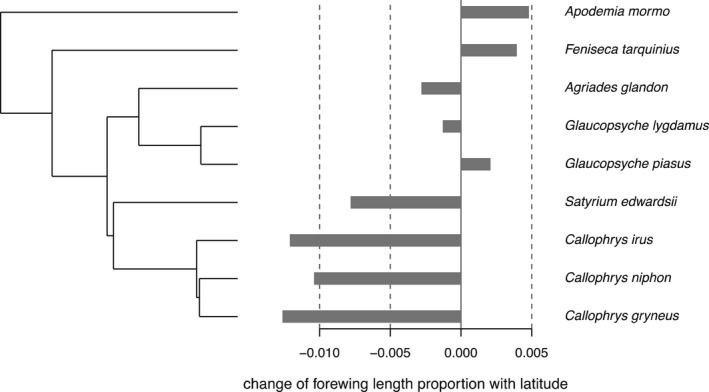
Phylogeny and bar plots of the change in the proportion of forewing length with latitude for each species

Using four metrics of phylogenetic signal (Moran's I, Abouheif's *C*_mean,_ Blomberg's K and K*, and Pagel's λ), we found that species that are more closely related to each other tend to have more similar body size–latitude relationships (Moran's *I* = 0.107, *p* = .011; Abouheif's *C*_mean_ = 0.473, *p* = .003; Blomberg's *K* = 1.933, *p* < .001; K* = 1.709, *p* < .001, and Pagel's *λ* = 1.106, *p* = .009; Figure 5).

### 3.4 Influence of host nitrogen on slope of body size with latitude

**FIGURE 6:**
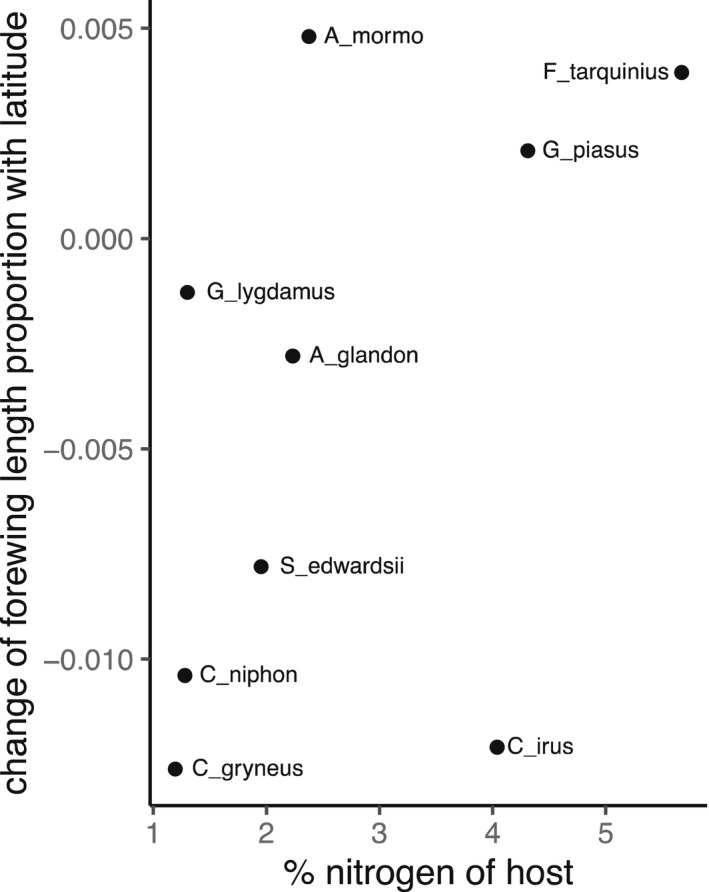
Scatter plot of change in proportion of forewing length with latitude against mean nitrogen content of caterpillar hosts. Points are labeled with their respective species abbreviations

We found no evidence that host nitrogen content was at all related to gradients of body size with latitude (*t* = 1.057, *p* = .326; Figure 6).

## 4 DISCUSSION

Latitudinal clines in body size among insect species are highly variable, leading some authors to contend that clines in even closely related species cannot be expected to be similar (Shelomi, 2012; Shelomi & Zeuss, 2017). At least one study on paper wasps (*Pollistes* spp.) supports this contention (Miller & Sheehan, 2021). By contrast, we found a significant phylogenetic signal by investigating latitudinal trends in eight lycaenid butterfly species and one species of the sister family, Riodinidae. Further, we hypothesized that species feeding on lower‐nitrogen hosts would have stronger converse‐Bergmann clines, but we found no evidence to support this hypothesis. However, some caution should be applied when interpreting our results, given our relatively small sample of these two families.

Our study supports previous authors’ findings that many butterflies exhibit negative relationships between body size and latitude, suggesting that the length of the growing season may limit the maximum size attained by adults in higher latitudes (Nygren et al., 2008; Nylin & Sviird, 1991). Four of nine species had significantly negative body‐size latitudinal clines, one had marginally negative body‐size latitudinal clines (*p* < .06), while the remaining four species had no significant relationship. Similarly, in a survey of 16 Swedish lycaenid and nymphalid butterflies, Nylin and Svard (1991) found that nine species had significantly negative body‐size clines, while the remaining seven species were not significant.

Latitudinal trends of body size in our study may hint at life history tradeoffs. Some empirical and theoretical research suggests that species may allocate more resources toward body size where growing seasons become prohibitively short for multiple generations, resulting in “sawtooth” patterns of latitudinal size variation (Nygren et al., 2008; Nylin & Sviird, 1991; Roff, 1980). Only two species in our study (*A*. *mormo* and *F*. *tarquinius*) have potentially shifting numbers of broods per year, and, perhaps not coincidentally, these two species display the most positive linear trends of body size with latitude. Visual examination of the latitudinal pattern of these species, however, provides less than convincing evidence for the hypothesized saw‐tooth pattern (Figure 4), although the inherent variability of these data may mask any such life history tradeoffs.

To our knowledge, ours is the first study to demonstrate a phylogenetic signal in body size latitudinal clines among invertebrate species, indicating that, for some taxa, shared underlying traits may contribute to this pattern. As previously mentioned, differences in shifting numbers of generations per year could contribute to latitudinal clines in body size. However, the phylogenetic signal in our study was likely driven by the surprisingly similar and strong converse‐Bergmann clines observed within the Eumaeini tribe (Figures 4 and Figure S1) despite their disparate life histories. For instance, *Callophrys niphon*, *C*.* gryneus*, and *Satyrium edwardsii*, feed on low‐nitrogen pine, juniper, and oak, respectively. In contrast, *C*. *irus* feeds on high‐nitrogen legumes. Further, Eumaeini in this study exhibit variation in voltinism (Table 1) and overwintering life stages—*S*. *edwardsii* overwinter as eggs, while the *Callophrys* spp. overwinter as pupae. Thus, it remains unclear what aspects of their biology make them particularly susceptible to decreased body size with higher latitudes and is an avenue worth further study. In contrast to the Eumaeini, *Glaucopsyche lygdamus* and *G*. *piasus* exhibited the least change in body size with latitude. In addition, they are also the two species of our study that are tended by ants. For this reason, it is interesting to speculate that ant associations may buffer the negative influence of shorter seasons in some way and, as ant associations likely have a phylogenetic signal themselves (Pierce et al., 2002), they may contribute to the phylogenetic pattern of latitudinal clines.

An interesting result of our study is that longitude, not latitude, was a strong driver of body size for non‐Eumaeini species. This result likely reflects underlying variation in abiotic or biotic factors and/or local adaptations that were not captured by mean annual temperature, precipitation, and elevation. For example, *A*. *mormo* appears to be particularly sensitive to longitudinal variation (Figures 3 and Figure S2), with longer‐winged individuals in the eastern part of its range. This sensitivity to longitude may reflect particularly strong among‐population genetic structure or even distinct subspecies within this taxon (Crawford et al., 2011; Proshek et al., 2013). Other species with distributions in western North America tended to decline in wing length toward the east. Studies with finer‐scale resolution and greater longitudinal overlap of ranges among species may provide interesting insights into drivers of these biogeographical trends.

Although we had hypothesized that nitrogen‐rich diets might reduce a species’ susceptibility to smaller body sizes at higher latitudes, we found no evidence for any relationship. This may be due in part to how we calculated mean host nitrogen content, which did not capture changes in host species with range, nor intraspecific variation within plant species, which is known to influence these trends for some species (Ho et al. 2010). Alternatively, shorter growing season length may not interact with lower dietary nitrogen to limit insects’ adult size.

The suggestion of a relationship between nitrogen and clines in body‐size with latitude was also proposed by Zeuss et al. (2017) to help explain the contrasting clines of mean interspecific body size between odonate and lepidopteran species assemblages in Europe; mean body size of univoltine odonate species assemblages increase with latitude, while mean body sizes of lepidopteran assemblages decrease. At higher latitudes, the greater abundance of coniferous plants, which tend to have lower nitrogen content, may account for the trends in mean body size among aggregations of lepidopterans in Europe. As noted by Zeuss et al. (2017), however, differences in aquatic and terrestrial habitats may also be a likely explanation for the distinction between odonates and lepidoptera.

Our study carries important implications for future work on biogeographic patterns in body size for invertebrates. Contrary to some other authors’ findings, we show that invertebrate taxa may have phylogenetic signals in body‐size gradients with latitude, although it appears to be at a particularly fine taxonomic scale (tribe, for lycaenids). Thus, authors attempting to use comparative studies to investigate drivers of these latitudinal trends will need to consider species’ evolutionary relationships to appropriately account for their traits’ nonindependence. Our results also undermined our hypothesis that nitrogen‐rich diets may ameliorate the decline of body‐size with latitude, and suggest that other as‐of‐yet unidentified ecophysiological factors are more important.

## AUTHOR CONTRIBUTIONS

**Andrew C. Merwin:** Conceptualization (lead); Data curation (equal); Formal analysis (equal); Investigation (lead); Methodology (equal); Project administration (lead); Resources (equal); Supervision (equal); Visualization (equal); Writing – original draft (lead); Writing – review & editing (equal). **Justin Hilliard:** Conceptualization (supporting); Data curation (equal); Formal analysis (equal); Investigation (equal); Methodology (equal); Project administration (equal); Supervision (equal); Visualization (equal); Writing – original draft (equal); Writing – review & editing (equal). **Ashley Larsen:** Conceptualization (supporting); Data curation (equal); Formal analysis (supporting); Investigation (equal); Methodology (supporting); Visualization (supporting); Writing – original draft (supporting); Writing – review & editing (equal). **Andrew G. Lasken:** Data curation (equal); Formal analysis (supporting); Investigation (supporting); Methodology (supporting); Project administration (supporting); Visualization (supporting); Writing – original draft (supporting); Writing – review & editing (equal). **Icesstrená Johnson:** Conceptualization (supporting); Data curation (equal); Formal analysis (supporting); Investigation (equal); Methodology (supporting); Visualization (supporting); Writing – original draft (supporting); Writing – review & editing (equal).

## CONFLICT OF INTEREST

The authors declare no conflict of interest.

## Supporting information

Supplementary Material

## Data Availability

We have provided the data and R code necessary to recreate our analyses and visualization at: https://doi.org/10.5281/zenodo.6478082 (Merwin et al., 2022).
