# Supplementary material for: Oh, the places you will grow: Intraspecific latitudinal clines in butterfly size suggest a phylogenetic signal
Source: Ecol Evol. 2022 May 19;12(5):e8913. doi: 10.1002/ece3.8913 (PMC9120895; doi:10.1002/ece3.8913)
Supplement: Supplementary file 1 — Supplementary Material [file ECE3-12-e8913-s001.docx]

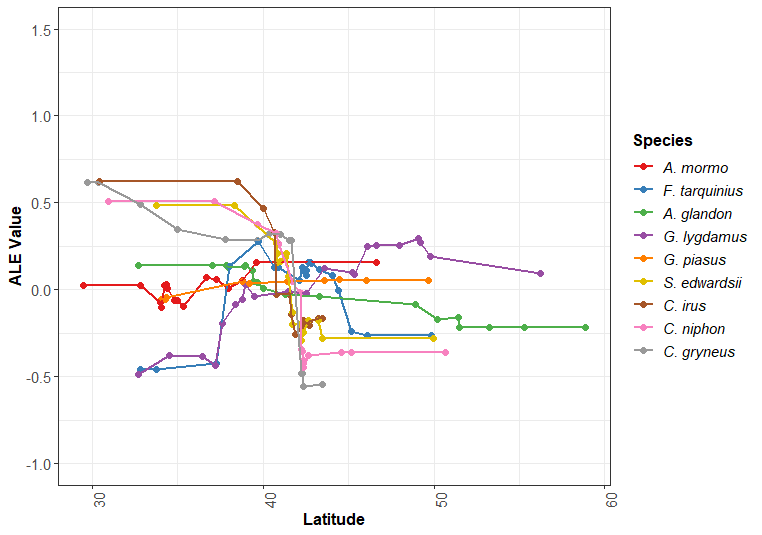


**Figure S1.** Accumulated Local Effects (ALE) plots of latitude as it affects butterfly forewing length for each species. Per species, quantiles are used to divide the covariate into intervals with equal sample numbers. The effect of each interval is calculated and compared against the overall average prediction, resulting in the ALE value for that interval. Every point represents the center of an interval and its associated ALE value. See Molnar (2019) for further reading. An interactive version of this plot is available in the Online Supplement. The interested reader is encouraged to explore that version where they can zoom in on regions and select which species to view.


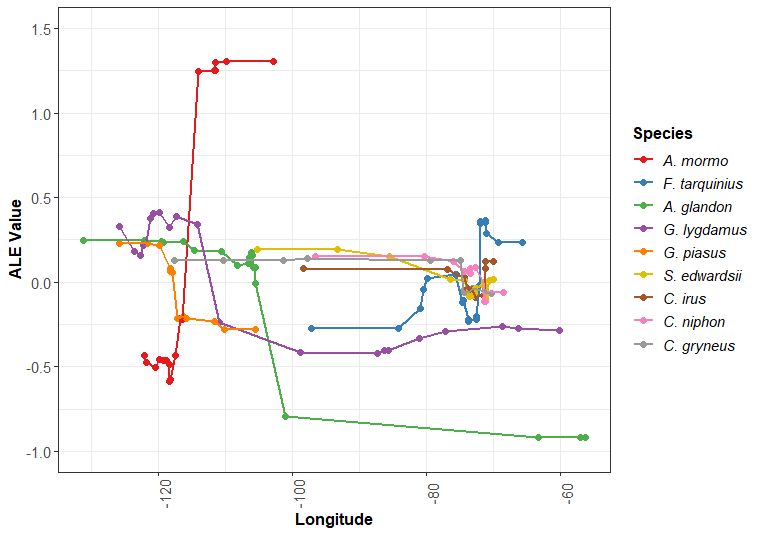


**Figure S2.** Accumulated Local Effects (ALE) plots of longitude as it affects butterfly forewing length for each species. Per species, quantiles are used to divide the covariate into intervals with equal sample numbers. The effect of each interval is calculated and compared against the overall average prediction, resulting in the ALE value for that interval. Every point represents the center of an interval and its associated ALE value. See Molnar (2019) for further reading. An interactive version of this plot is available in the Online Supplement. The interested reader is encouraged to explore that version where they can zoom in on regions and select which species to view.


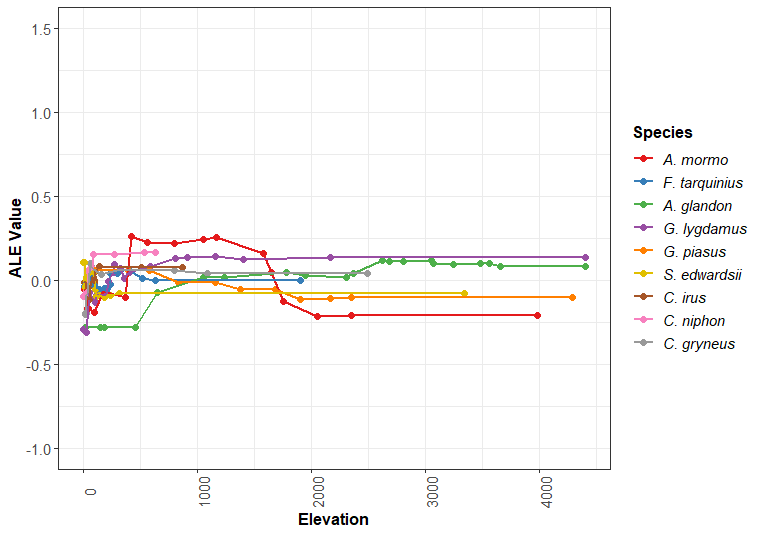


**Figure S3.** Accumulated Local Effects (ALE) plots of elevation as it affects butterfly forewing length for each species. Per species, quantiles are used to divide the covariate into intervals with equal sample numbers. The effect of each interval is calculated and compared against the overall average prediction, resulting in the ALE value for that interval. Every point represents the center of an interval and its associated ALE value. See Molnar (2019) for further reading. An interactive version of this plot is available in the Online Supplement. The interested reader is encouraged to explore that version where they can zoom in on regions and select which species to view.


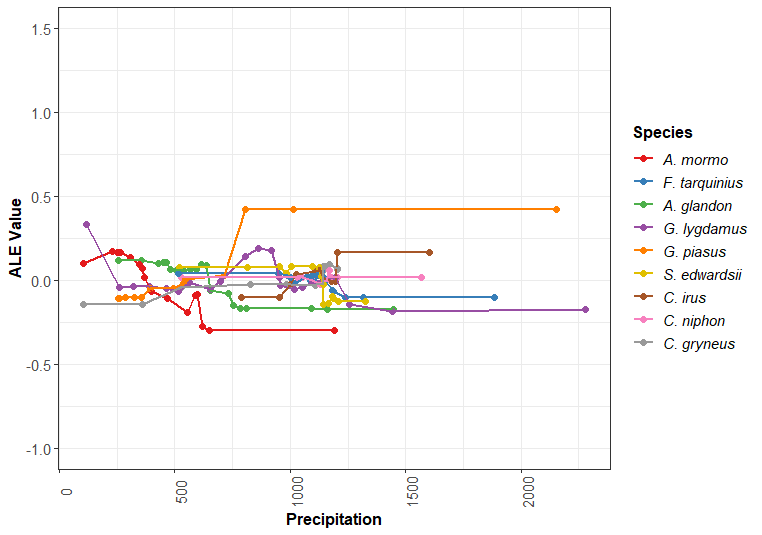


**Figure S4.** Accumulated Local Effects (ALE) plots of precipitation as it affects butterfly forewing length for each species. Per species, quantiles are used to divide the covariate into intervals with equal sample numbers. The effect of each interval is calculated and compared against the overall average prediction, resulting in the ALE value for that interval. Every point represents the center of an interval and its associated ALE value. See Molnar (2019) for further reading. An interactive version of this plot is available in the Online Supplement. The interested reader is encouraged to explore that version where they can zoom in on regions and select which species to view.


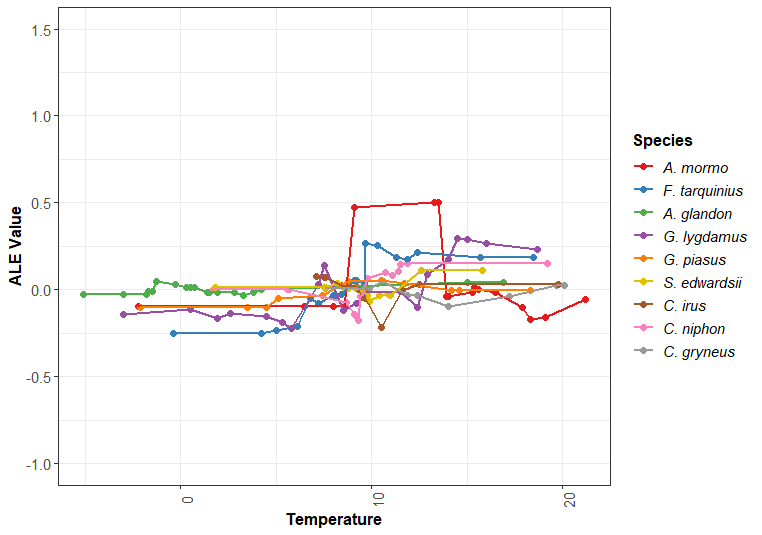


**Figure S5.** Accumulated Local Effects (ALE) plots of temperature as it affects butterfly forewing length for each species. Per species, quantiles are used to divide the covariate into intervals with equal sample numbers. The effect of each interval is calculated and compared against the overall average prediction, resulting in the ALE value for that interval. Every point represents the center of an interval and its associated ALE value. See Molnar (2019) for further reading. An interactive version of this plot is available in the Online Supplement. The interested reader is encouraged to explore that version where they can zoom in on regions and select which species to view.
